# Relationships Between Organic Acid Metabolism and the Accumulation of Sugars and Calcium in Fruits of *Cerasus humilis* During Different Development Stages

**DOI:** 10.3390/plants13213053

**Published:** 2024-10-31

**Authors:** Li Zhang, Jie Zhang, Jinli Guo

**Affiliations:** College of Horticulture and Plant Protection, Inner Mongolia Agricultural University, Hohhot 010010, China; zlkx628@163.com (L.Z.); g9616zj@163.com (J.Z.)

**Keywords:** *Cerasus humilis*, organic acid metabolism, sugar components, different forms of calcium, correlation analysis

## Abstract

*Cerasus humilis* fruit is known for its high acidity, surpassing that of most other fruits. The metabolism of organic acids in these fruits significantly influences sugar and calcium accumulation. However, research on this metabolic process is limited. This study investigates the organic acid metabolism and the accumulation patterns of sugars and calcium during the development of *Cerasus humilis* fruits. Using low−acid and high−acid varieties from Inner Mongolia, we compared organic acid components and the activity of relevant metabolic enzymes during fruit maturation. We also measured the content and proportions of various sugars and calcium forms, performing correlation analyses. Throughout the development and ripening of *Cerasus humilis* fruits, organic acids, sugars, and calcium exhibited consistent patterns of change across the two acidity types. Malic acid emerged as the most significant organic acid, while fructose was the primary sugar, and active calcium was the dominant calcium component. Correlation analyses indicated that malic acid and total acid positively correlated with sugar and water−soluble calcium content, negatively regulating other calcium forms. Conversely, NADP−ME, citric acid, and oxalic acid negatively correlated with sugars and water−soluble calcium, while positively affecting other calcium forms. In conclusion, the metabolism of organic acids during the development and maturation of *Cerasus humilis* fruits is closely linked to the accumulation of sugars and calcium. Malic acid, primarily regulated by NAD−MDH and NADP−ME, promotes the accumulation of sugars and water−soluble calcium but inhibits other calcium forms, while citric and oxalic acids inhibit sugar accumulation and promote non−water−soluble calcium forms.

## 1. Introduction

*Cerasus humilis* [*Cerasus humilis* (Bge.) Sok.], a member of the *Rosaceae* family and the subgenus *Cerasus*, is a unique fruit tree resource native to China [1]. It is primarily distributed in northern regions of China, including Shanxi, Inner Mongolia, Liaoning, and Hebei. The fruit is brightly colored, uniquely flavored, and rich in amino acids, vitamins, organic acids, and mineral elements such as calcium, iron, and magnesium. It can be consumed fresh or processed into products like fruit wine, juice, jam, red pigment, and nutritional supplements [2,3]. The calcium content in *Cerasus humilis* fruit is 2 to 10 times higher than that of other fruits and is easily absorbed by the human body, earning it the nickname “calcium fruit” [4]. Additionally, the seed of the *Cerasus humilis* can be used medicinally [5], and its leaves can be brewed into tea [6]. *Cerasus humilis* offers comprehensive utility through its fruit, leaves, and seeds. It is considered to possess potential health benefits, including antioxidant effects, digestive support, and immune enhancement. These attributes allow for its application across various fields, such as food and nutritional supplements. Consequently, *Cerasus humilis* has emerged as a fruit of significant commercial value, making it well-suited for further research and development [7,8].

Fruit quality encompasses both external appearance and internal nutritional attributes, with acidity and sugar serving as fundamental nutritional parameters [9]. Organic acids, present in all plants, play a crucial role in cellular metabolism. The primary organic acids found in fruits include malic acid, citric acid, oxalic acid, and succinic acid [10]. The principal sugars in fruits are fructose, sucrose, glucose, and sorbitol [11]. The content and ratio of various acids and sugars directly influence the taste and flavor of fruits, thereby determining their essential nutritional characteristics [12]. Organic acids not only directly affect fruit flavor but are also closely linked to overall nutritional quality and processing performance [13]. *Cerasus humilis* fruit is typically characterized by high acidity, with titratable acid content ranging from 1.0% to 2.0%, surpassing that of most other fruits. Consequently, acidity is a critical factor influencing the overall nutritional quality and processing capabilities of *Cerasus humilis* fruit [14]. There exists a correlation between the various acid and sugar components in fruits. Studies on hawthorn fruit have revealed a significant positive correlation between organic acid components and sugars [15]. Organic acids can combine with calcium to form calcium organic acids, enhancing calcium activity and promoting the absorption and utilization of calcium in the fruit [16]. As a fruit tree with notable acidity, the organic acid metabolism of *Cerasus humilis* inevitably affects the accumulation and transformation of sugars and calcium. However, research on the metabolism of organic acids and the accumulation of sugars and calcium in *Cerasus humilis* fruit remains limited.

*Cerasus humilis* is a malic acid−type fruit, with malic acid as the predominant organic acid. NAD−dependent malate dehydrogenase (NAD−MDH) and NADP−dependent malic enzyme (NADP−ME) are the key enzymes regulating the synthesis and degradation of malic acid, and their combined action determines the malic acid content in the fruit [17]. As a prominent fruit tree known for its calcium nutrition, *Cerasus humilis* has been the subject of our previous research, which indicated a close correlation between the levels of malic acid and the formation of calcium across various components [16]. The organic acids present in *Cerasus humilis* fruit are not limited to malic acid; they also include citric acid, succinic acid, and oxalic acid. However, the potential impact of varying concentrations and proportions of these acid components on the formation of sugar and calcium complexes in the fruit has yet to be reported. This study utilizes *Cerasus humilis* fruits with varying acidity levels as test materials. Through comparative analyses of the metabolic processes involving organic acids, sugar components, and different forms of calcium during the development and maturation of *Cerasus humilis* fruits, we explore the correlations between organic acid metabolism, sugar accumulation, and various forms of calcium. This research provides a foundation for further studies on quality control and enhancement of *Cerasus humilis* fruits, as well as innovations in resource utilization.

## 2. Results

### 2.1. Changes in Organic Acid Metabolism During the Development and Maturation of Cerasus humilis Fruits

#### 2.1.1. Changes in Organic Acid Content During the Development and Maturation of *Cerasus humilis* Fruits

**Figure 1:**
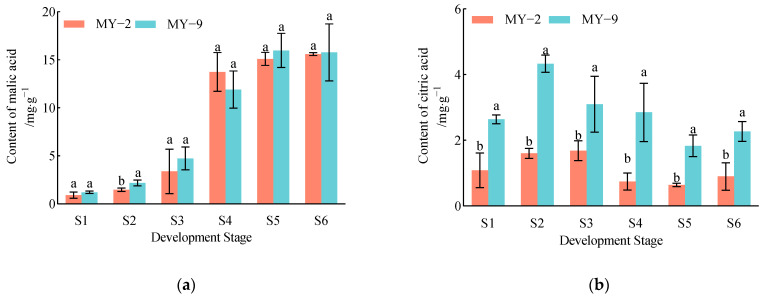

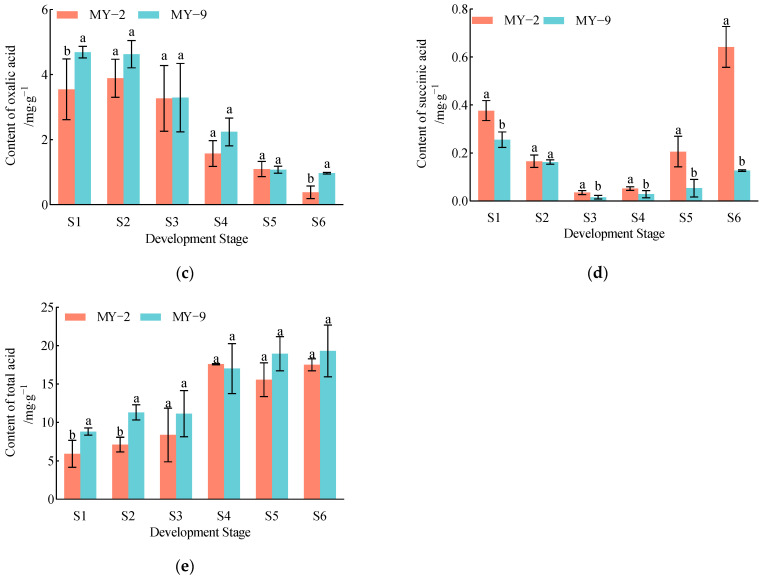
Changes in organic acid content during the development and maturation of *Cerasus humilis* fruits. (**a**) is the content of malic acid; (**b**) is the content of citric acid; (**c**) is the content of oxalic acid; (**d**) is the content of succinic acid; and (**e**) is the content of total acid. S1: young fruit stage; S2: pre−hard seed stage; S3: late hard seed stage; S4: coloring and enlargement stage; S5: hard−ripe stage; S6: fully ripe stage. Note: the lowercase letters indicate Duncan’s test results for *p* < 0.05 in the fruits of *Cerasus humilis* at different developmental stages for MY−2 and MY−9; the error bars represent the average standard deviation (*n* = 3).

During the development and maturation of *Cerasus humilis* fruits, the malic acid content exhibited a continuous increase in both low−acid and high−acid types. Initially, the malic acid content was extremely low, ranging from the young fruit stage to the late hard seed stage. It then rapidly increased during the coloring and enlargement stage, peaking at the fully ripe stage. Conversely, the citric acid content initially rose before declining, with the highest levels observed at the pre−hard seed stage, followed by a steady decrease. The oxalic acid content gradually decreased, reaching its lowest point at the fully ripe stage. The succinic acid content showed an initial decline, followed by an increase, with its lowest level recorded at the late hard seed stage. Throughout the development and maturation process, the malic acid content in fruits was significantly higher in the later stages (from the coloring and enlargement stage to full ripeness) compared to the earlier stages (from the young fruit stage to the late hard seed stage). In contrast, the citric acid and oxalic acid contents were greater in the earlier developmental stages (from the young fruit stage to the late hard seed stage) than in the later stages (from the coloring and enlargement stage to full ripeness). The total acid content in fruits with different acidity levels mirrored the trend of malic acid, displaying a continuous increase, with later developmental stages showing higher total acid levels than earlier stages (Figure 1). In summary, while the changes in the content of various organic acids during the development and maturation of *Cerasus humilis* fruits were consistent across different acidity levels, there were notable differences in the actual concentrations. High−acid *Cerasus humilis* fruits had higher total acid, malic acid, citric acid, and oxalic acid contents compared to low−acid fruits, with citric acid levels being significantly higher in the high−acid fruits. Conversely, succinic acid content was relatively higher in low−acid *Cerasus humilis* fruits.

#### 2.1.2. Changes in Organic Acid Metabolic Enzymes During the Development and Maturation of *Cerasus humilis* Fruits

**Figure 2:**
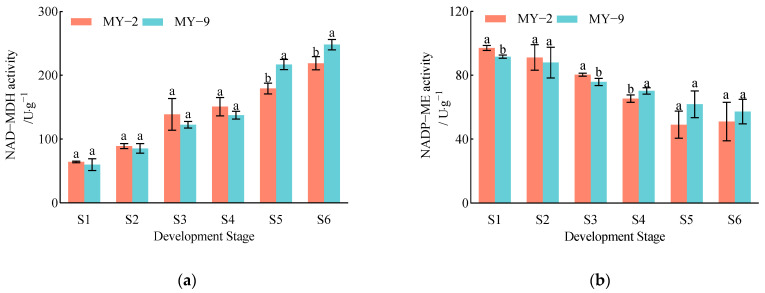
Changes in organic acid metabolic enzymes during the development and maturation of *Cerasus humilis* fruits. (**a**) is the NAD−MDH activity and (**b**) is the NADP−ME activity. S1: young fruit stage; S2: pre−hard seed stage; S3: late hard seed stage; S4: coloring and enlargement stage; S5: hard−ripe stage; S6: fully ripe stage. Note: the lowercase letters indicate Duncan’s test results for *p* < 0.05 in the fruits of *Cerasus humilis* at different developmental stages for MY−2 and MY−9; the error bars represent the average standard deviation (*n* = 3).

During the development and maturation of *Cerasus humilis* fruits, the activity of NAD−MDH in both low−acid and high−acid fruits exhibited a gradual increase. Enzyme activity was low in the early stages of fruit development, reaching its peak at the fully ripe stage, which aligns with the trend observed in malic acid content. Conversely, the activity of NADP−ME in both types of fruits exhibited a significant decline. NADP−ME activity was higher during the early stages of fruit development but decreased to its lowest level at the fully ripe stage, showing an opposite trend to that of malic acid content (Figure 2). In summary, the trends in the changes of organic acid metabolic enzymes were consistent across fruits of different acidity levels. NAD−MDH serves as the key enzyme for the synthesis of malic acid, while NADP−ME functions as the key enzyme for the degradation of malic acid.

#### 2.1.3. Changes in the Proportion of Organic Acid Components During the Development and Maturation of *Cerasus humilis* Fruits

**Figure 3:**
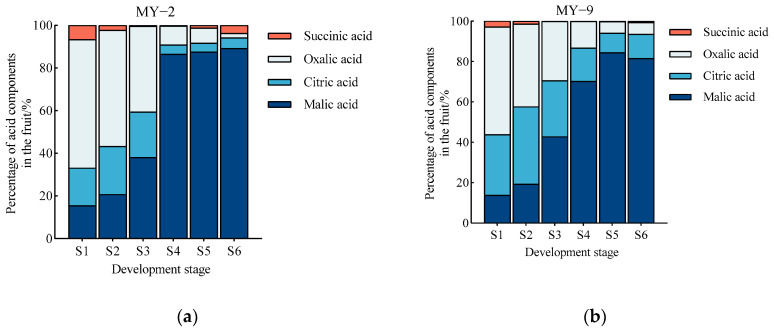
Changes in the proportions of organic acid components during the development and maturation of *Cerasus humilis* fruits. (**a**) is the percentage of acid components in low−acid *Cerasus humilis* MY−2 fruits and (**b**) is the percentage of acid components in high−acid *Cerasus humilis* MY−9 fruits. S1: young fruit stage; S2: pre−hard seed stage; S3: late hard seed stage; S4: coloring and enlargement stage; S5: hard−ripe stage; S6: fully ripe stage.

During the development and maturation of *Cerasus humilis* fruits, the proportion of malic acid content in both low−acid and high−acid fruits showed a gradual increase. This proportion was significantly higher in the later developmental stages compared to the earlier stages, reaching 89.10% and 81.54% at the fully ripe stage, respectively. In contrast, the proportion of citric acid and oxalic acid content exhibited a decreasing trend, with higher levels observed in the earlier stages compared to the later stages. At the fully ripe stage, the proportions of citric acid were 5.07% and 11.89%, while the proportions of oxalic acid were 2.15% and 5.90%, respectively. The proportion of succinic acid content demonstrated an initial decrease followed by an increase, with proportions at the fully ripe stage being 3.67% and 0.67%, the lowest among the organic acids (Figure 3). In summary, at the fully ripe stage, the proportion of malic acid in both low−acid and high−acid *Cerasus humilis* fruits exceeded 80%, confirming that *Cerasus humilis* is classified as a malic acid−type fruit. Additionally, the proportions of malic acid and succinic acid were higher in low−acid *Cerasus humilis* fruits compared to high−acid fruits, whereas the proportions of citric acid and oxalic acid were lower in low−acid fruits.

### 2.2. Changes in Sugar Accumulation During the Development and Maturation of Cerasus humilis Fruits

#### 2.2.1. Changes in Sugar Content During the Development and Maturation of *Cerasus humilis* Fruits

**Figure 4:**
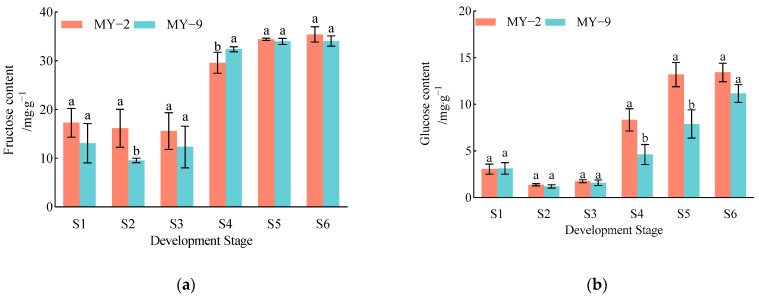

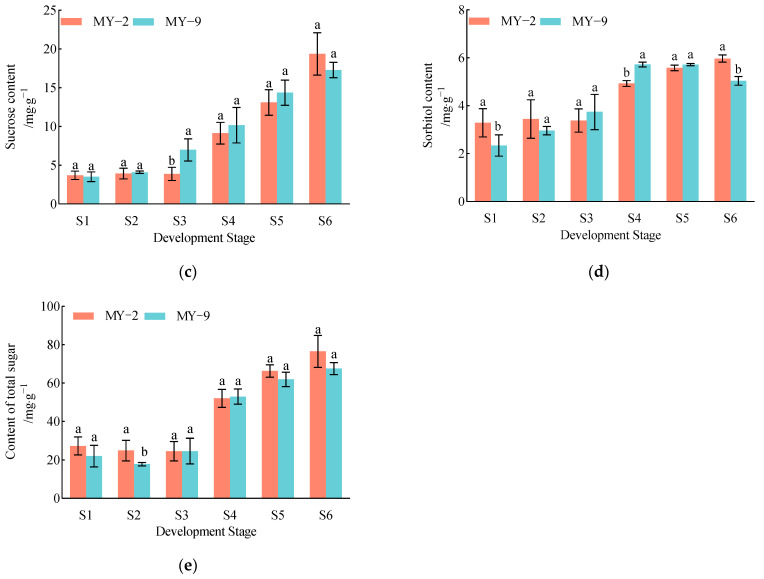
Changes in sugar content during the development and maturation of *Cerasus humilis* fruits. (**a**) is the fructose content; (**b**) is the glucose content; (**c**) is the sucrose content; (**d**) is the sorbitol content; and (**e**) is the content of total sugar. S1: young fruit stage; S2: pre−hard seed stage; S3: late hard seed stage; S4: coloring and enlargement stage; S5: hard−ripe stage; S6: fully ripe stage. Note: the lowercase letters indicate Duncan’s test results for *p* < 0.05 in the fruits of *Cerasus humilis* at different developmental stages for MY−2 and MY−9; the error bars represent the average standard deviation (*n* = 3).

During the development and maturation of *Cerasus humilis* fruits, the contents of fructose and glucose in both low−acid and high−acid fruits initially decreased slightly before showing an overall upward trend. These sugar contents were lower from the young fruit stage to the late hard seed stage, experiencing a slight decrease, followed by a rapid increase after the coloring and enlargement stage, ultimately reaching their highest levels at the fully ripe stage. Sucrose content displayed a gradual increase, remaining low during the early developmental stages and then rising sharply after the late hard seed stage, achieving its peak at the fully ripe stage. The trend for sorbitol content was less pronounced but also indicated an overall increase, with higher levels observed in the later stages of fruit development. Throughout the development and maturation process, the contents of fructose, glucose, sucrose, and sorbitol in fruits of varying acidity levels were significantly higher in the later developmental stages (from the coloring and enlargement stage to full ripeness) compared to the earlier stages (from the young fruit stage to the late hard seed stage). The changes in total sugar content mirrored those of fructose and glucose, showing a slight decrease in the early stages, followed by a rapid increase after the coloring and enlargement stage. The total sugar content in the later stages was greater than in the earlier stages (Figure 4). In summary, the changes in the content of various sugar components in *Cerasus humilis* fruits of different acidity levels were generally similar throughout the development and maturation process, with higher sugar content in the later stages of fruit development. Overall, low−acid *Cerasus humilis* fruits exhibited higher levels of fructose, sucrose, glucose, and sorbitol, with the most significant difference noted in glucose content between the two types of fruits.

#### 2.2.2. Changes in the Proportion of Sugar Components During the Development and Maturation of *Cerasus humilis* Fruits

**Figure 5:**
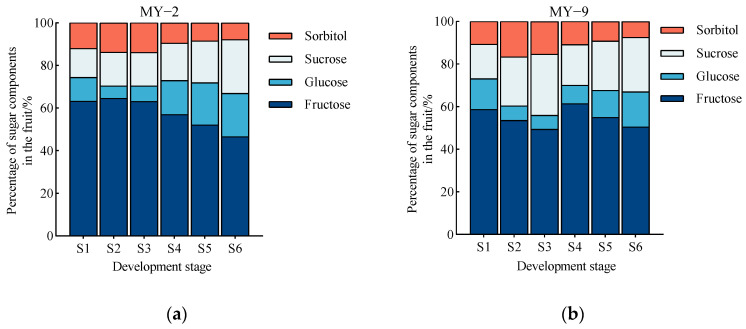
Changes in the proportions of sugar components during the development and maturation of *Cerasus humilis* fruits. (**a**) is the percentages of sugar components in low−acid *Cerasus humilis* MY−2 fruits; (**b**) is the percentages of sugar components in high−acid *Cerasus humilis* MY−9 fruits. S1: young fruit stage; S2: pre−hard seed stage; S3: late hard seed stage; S4: coloring and enlargement stage; S5: hard−ripe stage; S6: fully ripe stage.

During the development and maturation of *Cerasus humilis* fruits, the proportion of fructose content in both low−acid and high−acid fruits fluctuated between 46.52% and 63.21%. Throughout the developmental stages, fructose remained the primary sugar component, with proportions at the fully ripe stage recorded at 46.52% and 50.46%, respectively. The proportion of glucose initially decreased before subsequently increasing. It was lower during the early developmental stages but gradually rose after the coloring and enlargement stage, ultimately reaching its highest proportions at the fully ripe stage, with values of 20.38% and 16.50%, respectively. The proportion of sucrose content in fruits of different acidity levels displayed no clear trend, fluctuating between 14.60% and 28.73%. However, it was relatively higher in the later stages of fruit development, with proportions at the fully ripe stage being 25.25% and 25.58%, respectively. The proportion of sorbitol showed a trend of initially increasing followed by a decrease, with proportions at the fully ripe stage being 7.86% and 7.46%, respectively, making it the lowest among the sugars (Figure 5). In summary, the most significant sugar component in *Cerasus humilis* fruits is fructose, followed by sucrose and glucose, with sorbitol having the lowest proportion. In mature fruits, the proportions of glucose and sorbitol are slightly higher in low−acid *Cerasus humilis*, while the proportions of fructose and sucrose are higher in high−acid *Cerasus humilis*.

### 2.3. Changes in Calcium Accumulation During the Development and Maturation of Cerasus humilis Fruits

#### 2.3.1. Changes in Different Forms of Calcium Content During the Development and Maturation of *Cerasus humilis* Fruits

**Figure 6:**
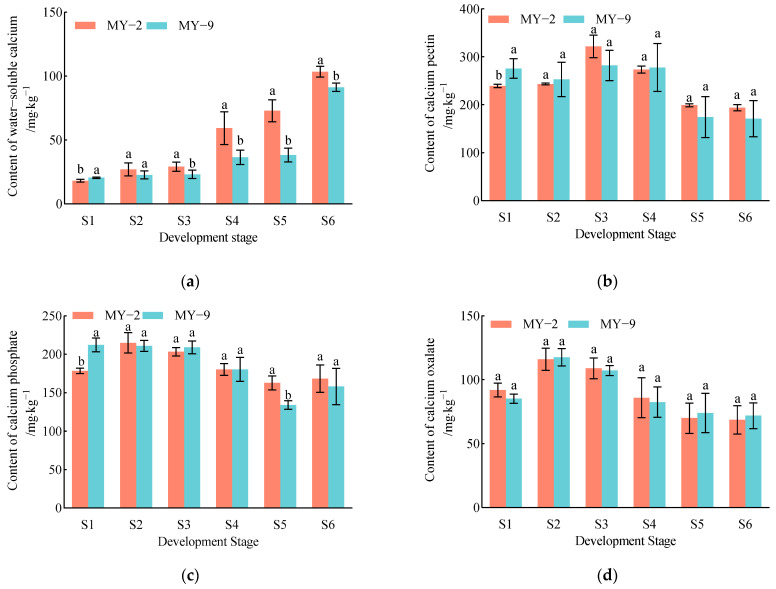

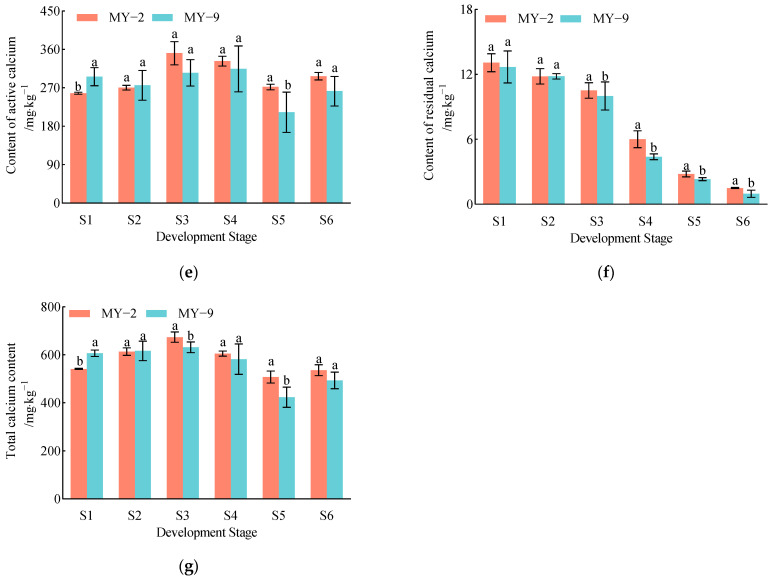
Changes in different forms of calcium content during the development and maturation of *Cerasus humilis* fruits. (**a**) is the content of water−soluble calcium; (**b**) is the content of calcium pectin; (**c**) is the content of calcium phosphate; (**d**) is the content of calcium oxalate; (**e**) is the content of active calcium; (**f**) is the content of residual calcium; and (**g**) is the content of total calcium. S1: young fruit stage; S2: pre−hard seed stage; S3: late hard seed stage; S4: coloring and enlargement stage; S5: hard−ripe stage; S6: fully ripe stage. Note: the lowercase letters indicate Duncan’s test results for *p* < 0.05 in the fruits of *Cerasus humilis* at different developmental stages for MY−2 and MY−9; the error bars represent the average standard deviation (*n* = 3).

During the development and maturation of *Cerasus humilis* fruits, the water−soluble calcium content in both low−acid and high−acid fruits exhibited a gradual increase, with the lowest levels recorded at the young fruit stage and the highest at the fully ripe stage. The contents of calcium pectin, calcium phosphate, calcium oxalate, and total calcium initially increased slightly from the young fruit stage to the hard seed stage, followed by a gradual decrease, reaching their lowest levels at the fully ripe stage. The content of active calcium did not display a clear trend, with the highest levels observed at the late hard seed stage and relatively lower levels at the fully ripe stage. Residual calcium content showed a steady decline, with only trace amounts remaining at the fully ripe stage (Figure 6). In summary, while the trends in the changes of different forms of calcium were similar across fruits of different acidity levels during development and maturation, differences in the actual content were observed. At the fully ripe stage, low−acid *Cerasus humilis* fruits contained higher levels of total calcium, water−soluble calcium, calcium pectin, calcium phosphate, active calcium, and residual calcium compared to high−acid *Cerasus humilis* fruits, whereas the content of calcium oxalate was lower in low−acid fruits.

#### 2.3.2. Changes in the Proportions of Calcium Components During the Development and Maturation of *Cerasus humilis* Fruits

**Figure 7:**
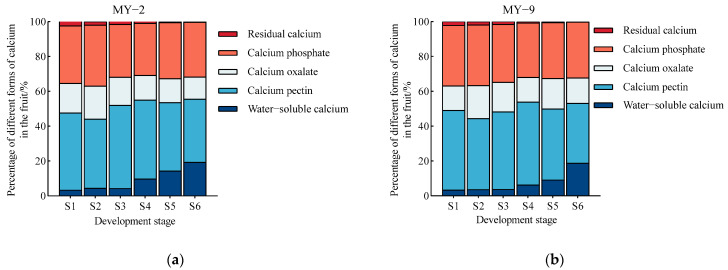
Changes in the proportions of calcium components during the development and maturation of *Cerasus humilis* fruits. (**a**) is the percentage of different forms of calcium in low−acid *Cerasus humilis* MY−2 fruits; and (**b**) is the percentage of different forms of calcium in high−acid *Cerasus humilis* MY−9 fruits. S1: young fruit stage; S2: pre−hard seed stage; S3: late hard seed stage; S4: coloring and enlargement stage; S5: hard−ripe stage; S6: fully ripe stage.

During the development and maturation of *Cerasus humilis* fruits, the proportion of water−soluble calcium content in both low−acid and high−acid fruits showed a gradual increase. This proportion was significantly higher in the later developmental stages (from the coloring and enlargement stage to the fully ripe stage) compared to the earlier stages (from the young fruit stage to the late hard seed stage), with the proportions at the fully ripe stage being 19.32% and 18.71%, respectively. In contrast, the proportion of calcium pectin content decreased as the fruits matured, with higher proportions observed in the earlier developmental stages compared to the later stages. At the fully ripe stage, the proportions of calcium pectin were 36.20% and 34.44%, respectively. Active calcium, which consists of water−soluble calcium and calcium pectin, accounted for 55.52% and 53.15% at the fully ripe stage. The proportions of calcium oxalate and calcium phosphate did not change significantly; at the fully ripe stage, the proportions of calcium oxalate were 12.79% and 5.90%, while the proportions of calcium phosphate were 31.41% and 32.08%, respectively. The proportion of residual calcium content gradually decreased, with the lowest proportions at the fully ripe stage being 0.28% and 0.20%, respectively (Figure 7). In summary, at the fully ripe stage, the proportion of active calcium in *Cerasus humilis* fruits of different acidity levels exceeded 50%, making it the most significant calcium component. This was followed by calcium pectin and calcium phosphate, with water−soluble calcium and calcium oxalate next in line, and residual calcium having the lowest proportion. In mature fruits, low−acid *Cerasus humilis* exhibited slightly higher proportions of water−soluble calcium and calcium pectin, while high−acid *Cerasus humilis* had higher proportions of calcium oxalate and calcium phosphate.

### 2.4. Correlations Between Organic Acid Metabolism and Sugars and Calcium During the Development and Maturation of Cerasus humilis Fruits

#### 2.4.1. Correlations Between Organic Acid Metabolism and Sugars During the Development and Maturation of *Cerasus humilis* Fruits

**Figure 8:**
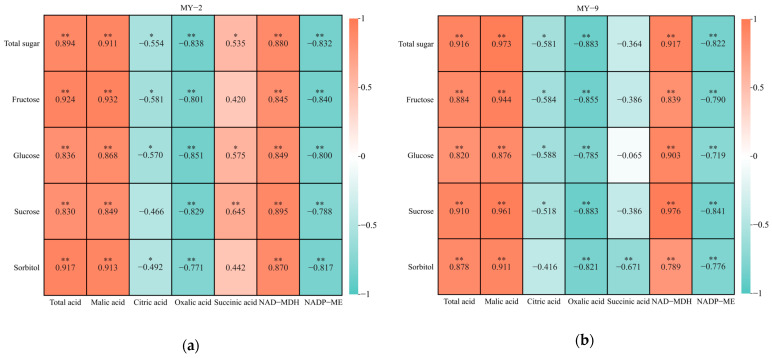
Correlations between organic acid metabolism and sugars during the development and maturation of *Cerasus humilis* fruits. “*” and “**” indicate significant and highly significant correlations at the *p* < 0.05 and *p* < 0.01 levels, and colored squares are heatmap representations of correlations, with orange squares representing positive correlations and green squares representing negative correlations. (**a**) is the correlation heatmap of low−acid *Cerasus humilis* MY−2; and (**b**) is the correlation heatmap of high−acid *Cerasus humilis* MY−9.

As *Cerasus humilis* fruits develop and mature, both the total acid and malic acid exhibit highly significant positive correlations with all sugar components and total sugar across fruits of different acidity levels. In contrast, citric acid demonstrates negative correlations with all sugar components and total sugar. Additionally, oxalic acid content shows highly significant negative correlations with all sugar components and total sugar. The correlations between succinic acid and sugar components vary between the two types of *Cerasus humilis*; in low−acid fruits, succinic acid has positive correlations with all sugar components and total sugar, while in high−acid fruits, it exhibits varying degrees of negative correlation. The correlations of NAD−MDH with all sugar components and total sugar aligns with those of malic acid, revealing highly significant positive correlations. Conversely, NADP−ME shows highly significant negative correlations with all sugar components and total sugar (Figure 8). In summary, malic acid, NAD−MDH, and total acid are positively correlated with the sugar components and total sugar. This suggests that as the activity of NAD−MDH increases, resulting in a rise in the synthesis of malic acid and total organic acids, the sugar content in the fruit also increases. On the other hand, NADP−ME, citric acid, and oxalic acid are negatively correlated with the sugar components and total sugar, indicating that these components negatively regulate sugar content in the fruit. Moreover, succinic acid positively regulates sugar content in low−acid *Cerasus humilis* while negatively regulating it in high−acid *Cerasus humilis*.

#### 2.4.2. Correlations Between Organic Acid Metabolism and Calcium During the Development and Maturation of *Cerasus humilis* Fruits

**Figure 9:**
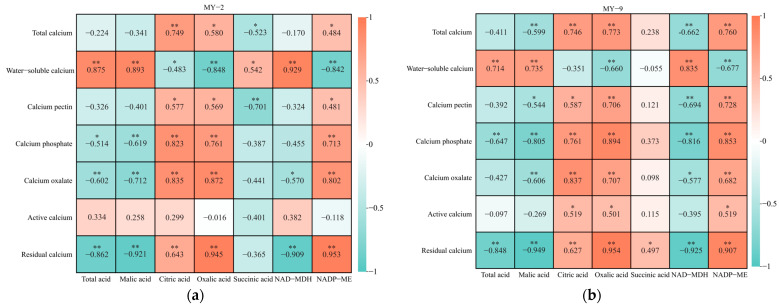
Correlations between organic acid metabolism and calcium during the development and maturation of *Cerasus humilis* fruits. “*” and “**” indicate significant and highly significant correlations at the *p* < 0.05 and *p* < 0.01 levels, and colored squares are heatmap representations of correlations, with orange squares representing positive correlations and green squares representing negative correlations. (**a**) is the correlation heatmap of low−acid *Cerasus humilis* MY−2; and (**b**) is the correlation heatmap of high−acid *Cerasus humilis* MY−9.

As *Cerasus humilis* fruits develop and mature, correlation analysis of the changes in various organic acid components and different forms of calcium reveals that, across fruits of different acidity levels, both malic acid and total acid have a highly significant positive correlation with water−soluble calcium, while exhibiting varying degrees of negative correlation with other forms of calcium and total calcium. Conversely, citric acid and oxalic acid display significant or highly significant negative correlations with water-soluble calcium and significant or highly significant positive correlations with other forms of calcium and total calcium. In low−acid *Cerasus humilis*, succinic acid shows a significant positive correlation with water−soluble calcium and varying degrees of negative correlation with other forms of calcium and total calcium. In high−acid *Cerasus humilis*, succinic acid is positively correlated with different forms of calcium and total calcium. NAD−MDH has a highly significant positive correlation with water−soluble calcium, along with varying degrees of negative correlation with other forms of calcium and total calcium. Conversely, NADP−ME exhibits a highly significant negative correlation with water−soluble calcium while positively correlating with other forms of calcium and total calcium (Figure 9). In summary, the correlations between malic acid, citric acid, oxalic acid, total acid, and related metabolic enzymes with different forms of calcium and total calcium are consistent in *Cerasus humilis* fruits of different acidity levels, although succinic acid shows slightly different but generally weaker correlations. Overall, malic acid and organic acids positively regulate water−soluble calcium while negatively regulating other forms of calcium in *Cerasus humilis* fruits. In contrast, citric acid and oxalic acid exhibit the opposite trend, negatively regulating water−soluble calcium while positively regulating other forms of calcium and total calcium.

## 3. Discussion

### 3.1. Changes in Organic Acid Metabolism During the Development and Maturation of Cerasus humilis Fruits

Organic acids are crucial factors affecting fruit quality, accumulating differently in various fruits as they develop [18]. In this study, four organic acids were detected in *Cerasus humilis* fruits: malic acid, citric acid, oxalic acid, and succinic acid. As the fruits matured, the total organic acid content in *Cerasus humilis* fruits of different acidity levels exhibited a gradual increase. The changes in malic acid content mirrored the trend in total acid, with both showing a consistent rise. Citric acid content initially increased and then decreased, while oxalic acid content gradually declined, displaying an opposite trend to that of total acid and malic acid. Succinic acid content first decreased and then increased, contrasting with the trend of citric acid. During fruit growth and development, the content of organic acids typically increases, followed by a decrease as the fruit transitions from growth to maturation [19,20]. In this study, malic acid and total acid in *Cerasus humilis* fruits increased rapidly after the coloring and enlargement stage. Although the increase was smaller during the maturation stage, it did not decline. This finding is consistent with the observations of Ye et al. [21], who reported that the malic acid content in *Cerasus humilis* fruits grew slowly during the coloration stage after fruit set and increased rapidly in the later stages of fruit maturation. They also noted that the contents of citric acid, succinic acid, and oxalic acid sharply decreased as the fruit matured, with a slight rebound at full maturity due to weaker degradation. This consistency with our results may explain the higher acidity observed in *Cerasus humilis* fruits.

Organic acid metabolism is a highly complex process regulated by multiple genes and their associated enzymes, and it is influenced by environmental factors and cultivar characteristics [22,23]. The results of this study indicate that as *Cerasus humilis* fruits develop and mature, the activity of NAD−MDH in fruits of different acidity levels shows a gradual increase, aligning with the trend in malic acid content. Conversely, the activity of NADP−ME exhibits a gradual decline, which is contrary to the trend in malic acid content. This pattern is consistent with the changes in organic acid metabolic enzyme activities observed in previous studies on nectarines [24] and sand pear fruits [25]. Previous research on organic acid metabolism in *Cerasus humilis* fruits has demonstrated that NAD−MDH is a key enzyme promoting the accumulation of malic acid, while NADP−ME plays a crucial role in the degradation of malic acid [26]. The conclusions of this study are consistent with these findings; the results indicate that NAD−MDH is involved in the synthesis of malic acid, while NADP−ME is associated with its degradation.

Organic acids are vital substances influencing fruit flavor and intrinsic quality. Variations in their components and content lead to distinctive flavors in different types of fruits, with the primary acid component differing among them. For instance, apples, pears, and peaches are primarily malic acid−based [27,28,29], while pomelo and apricot fruits are mainly citric acid-based [30,31]. Fruits can be classified into malic acid type, citric acid type, and tartaric acid type based on the type and content of organic acids present [32]. In this study, the analysis of the proportion of various organic acids in *Cerasus humilis* fruits indicates that they are malic acid-type fruits. In both low-acid and high-acid *Cerasus humilis* fruits, malic acid accounts for over 80% at the fully ripe stage, making it the predominant component. Citric acid ranks second, while oxalic acid and succinic acid have much lower proportions. These findings align with those observed in apples, sweet cherries, and loquats [27,33,34]. The study by Wang et al. [14] also demonstrated that malic acid is the predominant acid in *Cerasus humilis* fruits, with only a small amount of citric acid, which is consistent with the results of this study.

### 3.2. Changes and Accumulation Patterns of Sugars During the Development and Maturation of Cerasus humilis Fruits

Fruit quality largely depends on the types and quantities of sugars present. Extensive research has investigated the sugar components and accumulation patterns in various fruits, including apples [27], pears [28], peaches [29], pomelos [30], apricots [31], loquats [34], and goji berries [35]. In the above studies, the sugar composition of apple, sand pear, loquat, and goji berry fruits were found to be similar, with fructose as the dominant sugar. In contrast, the primary sugar in apricot, peach, and pomelo fruits is sucrose, followed by fructose and glucose. These studies indicate that sugar composition varies among fruits, with fructose and sucrose being the predominant sugars in most fruits, while glucose and sorbitol are found in lower concentrations. In this study, as *Cerasus humilis* fruits matured, the total sugar content exhibited a gradual upward trend. Fructose and glucose contents initially decreased during the early developmental stages but subsequently rose rapidly in later stages. Sucrose content consistently increased throughout development, showing minimal growth in the early stages followed by a substantial rise in later stages. Sorbitol content also demonstrated an overall upward trend. Ultimately, the total sugar and each sugar component peaked at the fully ripe stage. The accumulation of various sugar types in *Cerasus humilis* fruits primarily occurred during the later developmental stages, similar to the sugar accumulation characteristics observed in peaches [29] and apricots [31]. Research by Qu et al. [36] on fresh jujubes further corroborates that sugar accumulation mainly takes place in the later stages of fruit development. Wang et al. [14] noted that the total sugar content and each sugar component in *Cerasus humilis* fruits generally increased during development, with a marked rise during fruit maturation. Other studies in *Cerasus humilis* fruits have reported that during the green mature and coloration stages, the total sugar, glucose, and fructose contents increase slowly, accompanied by very low sucrose levels. However, during the commercial maturity stage, the content of all soluble sugars in *Cerasus humilis* fruits rises rapidly, reaching peak levels at full ripeness. The results of this study align with these findings [21]. Therefore, to enhance sugar content in *Cerasus humilis* fruits, technical interventions should focus on the later stages of fruit development.

The proportions of sugar components vary across different fruits. Previous studies have showed that apple fruits primarily accumulate monosaccharides, with fructose being the dominant soluble sugar, followed by sucrose and glucose. In sweet cherries, glucose is the most abundant sugar, followed by fructose [27]. For apricot fruits, the main soluble sugars are sucrose, fructose, and glucose, with sucrose being the most prevalent, along with a small amount of sorbitol [31]. In pear fruits, the primary sugars include fructose, glucose, sorbitol, and sucrose, with fructose being the most abundant, followed by glucose [28]. In this study, we analyzed the changes in the proportions of sugar components in *Cerasus humilis* fruits at different developmental stages. The results showed that, throughout development, fructose consistently remained the most dominant sugar, followed by sucrose and glucose, with sorbitol being the least abundant. This suggests that *Cerasus humilis* is a fructose−accumulating species, similar to apples, sand pears, loquats, and goji berries, where fructose is the predominant sugar. These findings align with those of Wang et al. [14], who reported that in the *Cerasus humilis* varieties Nongda 3, Nongda 4, and Nongda 5, fructose was the main sugar, with lower levels of sucrose and glucose. However, they contrast with the results of Ye et al. [21], who found that in the Jingou 1, Jingou 2, and Jingou 3 varieties, *Cerasus humilis* fruits are sucrose−accumulating. Additionally, Mo et al. [1] measured sugar component levels in 57 *Cerasus humilis* germplasms and found that the primary soluble sugars were sucrose, glucose, fructose, and sorbitol, with a higher sucrose content. These discrepancies may be attributed to variations in the characteristics of different *Cerasus humilis* varieties.

### 3.3. Changes and Accumulation Patterns of Calcium During the Development and Maturation of Cerasus humilis Fruits

Calcium is an essential nutrient for plant growth and development, playing a pivotal role in determining fruit quality, particularly during the later stages of fruit development. Calcium content directly influences fruit quality, post-harvest storage, and transportation [37]. In this study, we investigated the levels of total calcium, water−soluble calcium, calcium pectin, active calcium, calcium phosphate, calcium oxalate, and residual calcium at various developmental stages in low−acid and high−acid *Cerasus humilis* fruits. The results demonstrated that the contents of total calcium, calcium pectin, calcium phosphate, and calcium oxalate in *Cerasus humilis* fruits initially increased and then decreased, peaking at the pre−hard seed or late hard seed stages. Residual calcium content showed a continuous decline, whereas water−soluble calcium content gradually increased, reaching its maximum at the fully ripe stage. These findings align with previous research [38]. Calcium accumulation in *Cerasus humilis* fruits occurs mainly during the cell division and expansion stages. From the young fruit stage to the hard seed stage, calcium pectin accumulation dominates [39]. This is because large amounts of calcium are necessary for promoting the rapid growth of new cells, the development of the middle lamella between cells, and the formation of new cell walls. During the cell division stage, calcium pectin accumulates rapidly, with significant increases from the young fruit stage to the hard seed stage. As the fruit continues to mature and grow in size, transpiration efficiency decreases, limiting the calcium available for cell wall development. As a result, calcium absorption slows, and the relative calcium content begins to decline. During fruit maturation, water−soluble calcium becomes the dominant form. The rapid expansion of fruit cells, driven by vacuole enlargement, increases water−soluble calcium, which primarily accumulates in vacuoles [40]. Therefore, as the fruit reaches maturity, the content of water-soluble calcium steadily rises.

Calcium in fruits exists primarily in forms such as water−soluble calcium, calcium pectin, calcium phosphate, and calcium oxalate, though the proportions of these forms vary [41]. The results indicated that the trends in calcium form proportions were generally consistent between the two types of *Cerasus humilis*. Across the developmental stages, the predominant form of calcium in *Cerasus humilis* fruits was active calcium, followed by calcium pectin and calcium phosphate. Water−soluble calcium and calcium oxalate were less dominant, while residual calcium had the lowest proportion. Active calcium, comprising water-soluble and calcium pectin, plays a key role in the absorption and accumulation of calcium in *Cerasus humilis*. In this study, the proportion of active calcium in fully ripe fruits of both low-acid and high−acid *Cerasus humilis* exceeded 50%. In mature fruits, the proportions of water−soluble calcium and calcium pectin were slightly higher in low-acid *Cerasus humilis* compared to high−acid fruits. On the other hand, calcium oxalate and calcium phosphate were more abundant in high−acid *Cerasus humilis*. This variation may be attributed to the higher levels of organic acids in low−acid *Cerasus humilis*, which bind to calcium ions to form organic acid–calcium complexes that accumulate in vacuoles and are stored as water−soluble calcium. This process reduces organic acid levels and increases the content of active calcium.

### 3.4. Relationships Between Organic Acid Metabolism and Changes in Sugar and Calcium Accumulation During the Development and Maturation of Cerasus humilis Fruits

Organic acids, as key flavor compounds, have a significant impact on fruit quality and commercial value. Previous studies have demonstrated that organic acid content can influence other nutritional qualities of fruits [21]. In this study, two types of *Cerasus humilis* with different acidity levels were selected to investigate the relationship between organic acid metabolism and the main nutritional components—specifically, sugars and calcium during fruit development and maturation. The results revealed that malic acid and total acid, along with the key enzyme responsible for malic acid synthesis, NAD−MDH, were positively correlated with the sugar components and total sugar content in *Cerasus humilis* fruits. This suggests that as NAD−MDH activity increases, leading to higher malic acid content, the sugar content in the fruit also rises. In contrast, citric acid and oxalic acid exhibited negative correlations with sugar components and total sugar content, indicating that these acids may negatively regulate sugar accumulation in the fruit. Significant differences were observed in the levels of citric acid and oxalic acid between the two types of *Cerasus humilis*, with high−acid fruits containing significantly higher amounts of both acids at the fully ripe stage compared to low−acid fruits. Consequently, high−acid fruits had relatively lower sugar content. In low−acid *Cerasus humilis*, succinic acid was found to positively regulate sugar content, whereas in high-acid fruits, succinic acid negatively regulated sugar content. Furthermore, the succinic acid content was significantly higher in low−acid fruits than in high−acid fruits, suggesting that variations in citric acid, oxalic acid, and succinic acid levels are likely the primary factors contributing to the differences in sugar and acid content between the two types of fruits. The relationships between malic acid, citric acid, oxalic acid, total acid, and various forms of calcium, including total calcium, were consistent in both low−acid and high−acid *Cerasus humilis*. However, correlations involving succinic acid were slightly different and generally weaker. Notably, NAD−MDH, malic acid, and total acid positively regulated water−soluble calcium levels in the fruits while negatively regulating other calcium forms. In contrast, citric acid and oxalic acid exhibited the opposite effects, negatively regulating water−soluble calcium and positively influencing other calcium forms and total calcium. These findings are in agreement with previous research [16], but they offer new insights into the relationships between oxalic acid and different calcium forms. During the early stages of fruit development, oxalic acid content was relatively high, but it declined sharply after the hard pit stage, leading to a rapid decrease in calcium oxalate and other non−water−soluble forms of calcium in the later stages of fruit development.

## 4. Materials and Methods

### 4.1. Experimental Materials

This experiment utilized two selected *Cerasus humilis* types: ‘Mengyou No.2’ (MY−2), characterized by relatively low fruit acidity, and ‘Mengyou No.9’ (MY−9), characterized by relatively high fruit acidity. These materials were planted at the *Cerasus humilis* research base of Inner Mongolia Agricultural University, located in Hohhot, Inner Mongolia, at a longitude of 111.65° and a latitude of 40.82°.

The growing season for *Cerasus humilis* spans from April to September, with an average temperature ranging from 12.6 °C to 22 °C and total precipitation amounting to 242.1 mm. For each type of *Cerasus humilis*, ten robust plants were selected and tagged. Each plant was retained with two fruiting branches and six vegetative branches, ensuring consistent cultivation management practices. During the flowering period (20 April), a foliar application of a 0.2% borax solution was administered once. In the fruit coloring and swelling stage, two applications of a 0.3% monopotassium phosphate solution were performed. Irrigation was conducted according to soil moisture conditions, ensuring timely watering during the budding phase (beginning in early April), the fruit swelling phase (starting in early August), and prior to the soil freeze. Watering was controlled during the flowering period, and irrigation was halted 7 to 10 days before fruit maturity.

### 4.2. Sample Collection

The experiment on *Cerasus humilis* entered the flowering period on 20 April 2023, with sample collection occurring from June to September 2023. Samples were collected at six stages: 50 days post-flowering on 10 June (designated as the young fruit stage S1), 80 days post-flowering on 10 July (designated as the pre−hard seed stage S2), 100 days post-flowering on 30 July (designated as the late hard seed stage S3), 120 days post-flowering on 20 August (designated as the coloring and enlargement stage S4), 135 days post-flowering on 5 September (designated as the hard−ripe stage S5), and 150 days post-flowering on 20 September (designated as the fully ripe stage S6). A total of 300 fruit samples were collected from the two types of *Cerasus humilis*, ensuring uniformity and the absence of pests and diseases, as well as no mechanical damage. For each sampling period, five fruits were selected from each of the upper, middle, and lower positions of each fruiting branch across 10 plants, resulting in a total of 20 fruiting branches. The collected fruits were mixed uniformly and divided into three portions. After washing with distilled water and air−drying at room temperature, they were rapidly frozen in liquid nitrogen and stored in a −80 °C freezer until all samples were collected for subsequent data analysis.

### 4.3. Experimental Index Measurement

#### 4.3.1. Measurement of Organic Acid Content and Related Metabolic Enzymes in *Cerasus humilis* Fruits

The organic acid content was measured following the method described by Ji et al. [42]. Approximately 5.0 g of *Cerasus humilis* fruit at different developmental stages was weighed and homogenized with 25 mL of 80% ethanol. The homogenate was extracted in a 75 °C water bath for 60 min, then filtered. The filtrate was evaporated to dryness at 60 °C using a rotary evaporator. The residue was dissolved in 3 mL of double-distilled water and filtered through a 0.45 μm membrane. The concentrations of malic acid, citric acid, oxalic acid, and succinic acid in fruit samples were quantified using high–performance liquid chromatography (HPLC) with a Waters E2695 separations module coupled to a 2489 UV/Visible detector (Waters Corporation, Milford, MA, USA).

HPLC conditions: Chromatographic separation was carried out on an Ultimate LP–C18 column (4.6 × 300 mm, 5 μm). The mobile phase consisted of 0.01 mol/L potassium dihydrogen phosphate aqueous solution and methanol (95:5, *v*/*v*). The column temperature was maintained at 30 °C, with a flow rate of 0.5 mL/min. A 10 μL aliquot was injected for each run, and detection was performed at a wavelength of 210 nm. The total run time for each analysis was 30 min.

Total acid = Malic acid + Citric acid + Oxalic acid + Succinic acid(1)

The activity of malic enzyme (NADP−ME) was measured using the NADP-ME activity assay kit [43] (Beijing solarbio science & technology Co., Ltd., Beijing, China), and the activity of malate dehydrogenase (NAD−MDH) was measured using the NAD-MDH activity assay kit [44] (Beijing solarbio science & technology Co., Ltd., Beijing, China). Each measurement was repeated three times.

#### 4.3.2. Measurement of Sugar Content in *Cerasus humilis* Fruits

The contents of fructose, glucose, and sucrose were measured based on the method described by Sheng et al. [45], with slight modifications.

For each developmental stage, 0.5 g of *Cerasus humilis* fruit was homogenized with 4 mL of 80% ethanol. The homogenate was incubated at 80 °C in a water bath for 30 min. After cooling, the mixture was centrifuged at 4000× *g* for 5 min, and the supernatant was collected. The residue was re–extracted twice with 80% ethanol, and the supernatants were combined. To the pooled supernatant, 50 mg of activated carbon was added for decolorization at 80 °C for 30 min, followed by centrifugation at 4000× *g* for 3 min. The final supernatant was adjusted to a volume of 10 mL.

Fructose determination: A 1 mL aliquot of the extract was mixed with 2.5 mL of anthrone–sulfuric acid reagent (prepared by dissolving 150 mg of anthrone in 72% sulfuric acid; freshly prepared before use). The mixture was incubated at 40 °C for 10 min, then rapidly cooled. After 10 min of color development, the absorbance at 620 nm (OD620) was measured using a UV–Vis spectrophotometer (UV8000, Shanghai Yuanxi Instrument Co., Ltd., Shanghai, China). A blank was prepared using 80% ethanol instead of the extract, and a 100 mg/L standard fructose solution was used as a reference.

Glucose determination: A 1 mL aliquot of enzyme solution (prepared by dissolving 10 mg o-dianisidine–HCl and 10 mg horseradish peroxidase, along with 0.1 mL of 100 U/mL glucose oxidase, and adjusting the final volume to 100 mL with water) was incubated in a 30 °C water bath for 5 min. Afterward, 1 mL of the extract was added, and the mixture was shaken to ensure thorough mixing. The reaction proceeded for 5 min, after which 2 mL of 10 mol/L sulfuric acid was added to terminate the reaction. The absorbance at 525 nm (OD525) was measured using a UV–Vis spectrophotometer (UV8000, Shanghai Yuanxi Instrument Co., Ltd.). A blank was prepared by replacing the extract with 80% ethanol, and a 100 mg/L standard fructose solution was used as a reference.

Sucrose determination: A 0.5 mL aliquot of the extract was mixed with 50 μL of 2 mol/L NaOH and incubated at 90 °C for 5 min. After rapid cooling, 2.5 mL of anthrone–sulfuric acid reagent (prepared by dissolving 150 mg of anthrone in 72% sulfuric acid; freshly prepared before use) was added, and the mixture was thoroughly mixed. The solution was incubated at 80 °C for 10 min, followed by rapid cooling. After 10 min of color development, the absorbance at 620 nm (OD620) was measured using a UV–Vis spectrophotometer (UV8000, Shanghai Yuanxi Instrument Co., Ltd.). A blank was prepared by replacing the extract with 80% ethanol, and a 100 mg/L standard sucrose solution was used as a reference.

Sorbitol content was determined using the Sorbitol Content Assay Kit [46] (Beijing solarbio science & technology Co., Ltd., Beijing, China). Each measurement was repeated three times.

Total sugar = Fructose + Glucose + Sucrose + Sorbitol(2)

#### 4.3.3. Measurement of Calcium Content in *Cerasus humilis* Fruits

The extraction of different forms of calcium was conducted according to the method described by Huang et al. [47]. Samples of 1.0 g of *Cerasus humilis* at different developmental stages were sequentially extracted using ultra–pure water, 1 mol/L sodium chloride, 2% acetic acid, and 5% hydrochloric acid in a water bath at 25 °C. The calcium extracted with ultra–pure water was classified as water–soluble calcium, the calcium extracted with sodium chloride was identified as calcium pectin, the calcium extracted with acetic acid was classified as calcium phosphate, and the calcium extracted with hydrochloric acid was identified as calcium oxalate. Finally, the remaining residue was digested using a mixed acid of HNO₃–HClO₄ (5:1) to determine the residual calcium content. The concentrations of the various forms of calcium were measured using a flame atomic absorption spectrophotometer (Hitachi ZA3000 series, Hitachi Scientific Instruments (Beijing) Co., Ltd., Beijing, China). Each measurement was repeated three times.

Active calcium = Water-soluble calcium + Calcium pectin(3)

Total calcium = Water-soluble calcium + Calcium pectin + Calcium phosphate + Calcium oxalate + Residual calcium(4)

### 4.4. Statistical Analysis

In the data analysis, Microsoft Excel 2010 and Graphpad Prism 8.0.2 were utilized for data processing and the creation of bar charts, while SPSS 25.0 was employed for statistical analysis and Pearson’s correlation analysis. The significance of differences was examined using ANOVA analysis and Duncan’s multiple comparisons with a significance level of *p* < 0.05. The correlation heat map was drawn using ChiPlot (https://www.chiplot.online/) (accessed on 18 August 2024)

## 5. Conclusions

In conclusion, *Cerasus humilis* fruits are characterized by being malic acid–dominant and fructose–accumulating. The metabolism of organic acids in these fruits is closely linked to the accumulation of sugars and calcium. Enhanced malic acid synthesis promotes an increase in sugar content and water–soluble calcium, while inhibiting the accumulation of other forms of calcium. Conversely, increased levels of citric and oxalic acids suppress sugar accumulation but promote the deposition of non–water–soluble calcium forms, such as calcium pectin, calcium phosphate, and calcium oxalate. In practical cultivation, regulating the metabolism of organic acids in *Cerasus humilis* fruit allows for effective management of sugar and calcium content. This approach not only provides a theoretical basis for enhancing the overall quality of *Cerasus humilis* but also offers practical guidance for farmers to optimize fertilization and management strategies. By modulating malic acid synthesis, it is possible to promote sugar accumulation and water–soluble calcium formation, which is particularly suitable for developing fresh–market *Cerasus humilis* varieties. Conversely, by regulating the synthesis of citric and oxalic acids, it is possible to lower sugar levels in the fruit while promoting the accumulation of non–water–soluble and total calcium, thereby facilitating the cultivation of low–sugar, high–calcium varieties suitable for processing. Moreover, further investigation into how organic acid metabolism influences the quality characteristics of fruit will provide new insights and directions for future breeding and cultivation innovations.

## Data Availability

Data are contained within the article.
